# Bioelectric Applications for Treatment of Melanoma

**DOI:** 10.3390/cancers2031731

**Published:** 2010-09-27

**Authors:** Stephen J. Beebe, Karl H. Schoenbach, Richard Heller

**Affiliations:** Frank Reidy Research Center for Bioelectrics/Old Dominion University 4211 Monarch Way, Suite 300, Norfolk, Virginia 23508, USA; E-Mails: kschoenb@odu.edu (K.H.S.); rheller@odu.edu (R.H.)

**Keywords:** electric fields, electroporation, gene delivery, cytokines, enhanced immunity, nanosecond pulsed electric fields, non-thermal effects, apoptosis, caspases, anti-angiogenesis

## Abstract

Two new cancer therapies apply bioelectric principles. These methods target tumor structures locally and function by applying millisecond electric fields to deliver plasmid DNA encoding cytokines using electrogene transfer (EGT) or by applying rapid rise-time nanosecond pulsed electric fields (nsPEFs). EGT has been used to locally deliver cytokines such as IL-12 to activate an immune response, resulting in bystander effects. NsPEFs locally induce apoptosis-like effects and affect vascular networks, both promoting tumor demise and restoration of normal vascular homeostasis. EGT with IL-12 is in melanoma clinical trials and nsPEFs are used in models with B16F10 melanoma *in vitro* and in mice. Applications of bioelectrics, using conventional electroporation and extensions of it, provide effective alternative therapies for melanoma.

## 1. Introduction

The skin is our largest organ and it is protected by melanocytes, which reside in the basal layer of the epidermis, where they produce melanin. Keratinocytes regulate melanocyte number, differentiation and melanin production in response to UV radiation. Perhaps because melanocytes have an inherent resilience to protect the skin, they cause one of the most deadly forms of skin cancers when they undergo tumorogenesis, and, because they originate in the neural crest and migrate to the skin with an innate program for motility, they may be more prone to metastasis. Although genetics plays a role in the development of melanoma, exposure to extreme sunlight (UVA and UVB) or tanning bed light (mostly UVA) is a dominant etiology for development of melanoma. The American Cancer Society estimated for 2009 that in the US, 68,720 new cases of melanoma (188 new cases/day) will be diagnosed and 8,650 people will die from the disease (24 melanoma deaths/day). Thus, the incidence of melanoma continues to increase and is a significant cause of morbidity and mortality in the Western world. Thus, metastatic melanoma remains a persistent therapeutic challenge. Successes in preventing this often fatal disease are limited and there are even fewer successes in developing a cure.

Effective treatment options for melanoma are significantly limited, especially for metastatic melanoma. Treatments include resection, when the disease is limited and localized, chemotherapy, radiation and/or immunotherapy. Chemotherapy approaches include DTIC (dacarbazine), alone or in combination with BCNU (carmustine) or cisplatin. The “Dartmouth regimen” includes these three drugs with tamoxifen. Another combination includes cisplatin, DTIC and vinblastin [1,2]. Addition of systemic immunotherapy with IL-2 and/or INFά to these regiments resulted in increased toxicity without improving progression-free or overall survival [3]. Unfortunately, metastatic melanoma is one of the most resistant cancers to a wide range of treatment modalities including single-agent and combination chemotherapy, immunotherapy, chemoimmunotherapy and a host of immune stimulators [4]. Since these therapies are wanting, novel approaches for treatments are need.

A major obstacle in treatments for cancer is that it is not a single disease, but hundreds of them; so even melanomas are not all alike. Cancers exhibit hundreds of genotypes defined by substantial numbers of mutations. To provide more focused characterization of cancer and in order to manage this array of diseases, Hanahan and Weinberg [5] defined six major hallmarks of cancer that exhibit physiological anomalies that control cell homeostasis and proliferation. These include self-sufficiency in growth signals, insensitivity to growth-inhibitory (antigrowth) signals, evasion of apoptosis, limitless replicative potential, sustained angiogenesis, and tissue invasion and metastasis. Kroemer and Pouyssegur [6] included evasion of immune surveillance as a seventh hallmark and Luo *et al*. [7] expanded these classic hallmarks to include stress phenotypes of tumorigenesis and defined a large class of non-oncogenes that are essential for cancer cell survival. These hallmarks provide major strategies to design targeted therapies for cancer treatment. Since there are many molecular mechanisms of cancer and they differ among cancer types, to design treatments for them, it is important to understand the pathobiology of each one. Over the last several years significant progress has been made to understand the pathobiology of melanoma and a number of treatments have been designed based on this knowledge.

In the last ten years, a new field of Bioelectrics has emerged with new therapeutic strategies to treat cancer using electric fields. Two different approaches using electric fields include those with durations that are in the milli-or micro-second range and intensities of V/cm. These include conditions that are referred to as conventional electroporation [8]. These pulses increase the permeability of the plasma membrane but have minimal effects on intracellular membranes. This review will discuss uses of conventional electroporation for the delivery of genes or electrogene therapy directed towards the hallmarks of cancer [5,6] to treat melanoma as a model for cancer. More recently, pulse power technology has given rise to electric field pulses that are in the sub-microsecond range, primarily nanosecond pulsed electric field (nsPEFs), with intensities of tens to hundreds of kV/cm. These pulses include conditions referred to as supra-electroporation causing high density nanopore formation in all cell membranes [9]. The role that these plasma membrane and intracellular membrane nanopores play in nsPEF effects remains to be determined. These pulses will be discussed here to eliminate melanoma through effects on several cancer hallmarks. However, before these technologies are discussed, other treatments for melanoma will be presented describing why new and novel strategies are need to treat melanoma.

### Targeted Therapies for Melanoma

Targeted therapies have been designed to attack specific mechanisms that cancers evade, especially those related to proliferation, apoptosis, angiogenesis and immune surveillance. Many cancer mutations occur in oncogenes and tumor suppressor genes in several major signaling pathways causing aberrant behavior in melanocytes resulting in melanoma. While there are four main clinical subtypes of melanoma (nodular, acral lentiginous, lentigo maligna and superficial spreading), none of these have been specifically associated with abnormal signaling mechanisms. Whereas it is known that there are mutations among these pathways that drive progression of cancer from normal through naevi, radial and vertical growth phases, it is not yet clear how these mutations and which ones affect signaling mechanisms to achieve progression and metastatic outcome. Major targeted treatment efforts have focused on mutations in several hyper-activated pathways in metastatic melanoma [2,10,11]. Two of these are pathways downstream of receptor tyrosine kinases, cytokines and G-protein coupled receptors that branch from one of three RAS genes (NRAS, HRAS and KRAS). The mutant small GTPases from these genes hyper-activate Raf/MEK/ERK signaling, which leads to survival and proliferation and PI(3)K/Akt/mTor signaling, which leads to cell survival, proliferation, growth and motility. Significant numbers of mutations have been identified in melanoma patients in these pathways including NRAS (15–30%), BRAF (50–70%), AKT3 (60%) and PTEN (5–20%), which inhibits PI(3)K signaling. 

There have been a number of drugs that target some of these mutant pathways. These include inhibitors of Ras, receptor tyrosine kinases, RAF, MEK, proteases, PI(3)Kinase, Akt, and mTOR. While some of these approaches are still in phase I or II clinical trials, some targeted drugs in the presence or absence of chemotherapeutic agents may be necessary. One such drug for metastatic melanoma that showed initial promise is PLX4032, a B Raf inhibitor [2]. The drug demonstrated efficacy only in patients with the BRAF mutation. PLX4032 showed both tumor shrinkage and delay in tumor progression in patients with the BRAF mutation and reports of improvement in clinical symptom. Partial responses have been observed in 70% of patients (greater than 30% tumor regression by Response Evaluation Criteria in Solid Tumors), minor responses in other patients (regression greater than 10% but less than 30%), disease control lasting up to 14 months with continuous therapy and interim median progression-free survival of at least six months. Regression of metastatic lesions was observed in all common metastatic sites (liver, lung and bone). Oral administration of the drug is relatively well tolerated with minor side effects. However, after chronic treatment, serious adverse events were observed in some patients, including possibly drug-related cutaneous squamous cell carcinoma, which is typically excised by a patient's dermatologist. While several patients in the trials have relapsed, many are still in remission. Nevertheless, this represents an important new therapeutic development in the treatment of melanoma. For those who experienced relapses, a second mutation appears to continue to drive tumorigenesis. It will be important to determine what this mutation is. In a multicenter phase III trial, approximately 700 previously untreated melanoma patients who will be randomized one-to-one with PLX4032 (960 mg BID) or dacarbazine (DTIC), a drug approved for the treatment of metastatic melanoma.

Another protein that may be worthy to target is STAT3 or signaling pathway upstream of STAT3 [12,13,14,15]. STAT3 is activated in 50-90% of cancers including a majority of melanoma cell lines and tumor samples tested [12,13]. It is downstream of several tyrosine kinases including SRC. STAT3 plays central roles in most cancer hallmarks including tumor cell survival, proliferation, angiogenesis, metastasis, and immune evasion. It drives or inhibits the expression of a wide variety of proteins that promote these functions [14]. Further, blockade of SRC or STAT3 induces apoptosis and tumor regression [12,15]. This protein could be an excellent target for small-molecule drugs to treat melanoma.

A mentioned earlier, melanomas are particularly resistant to apoptosis induction and are notoriously resistant to chemotherapeutic agents that induce apoptosis, suggesting a connection between the two [11,16,17]. Melanomas exhibit mutations that activate anti-apoptosis factors and/or inhibit pro-apoptotic factors. The BCL2 protein, which inhibits mitochondria-mediated apoptosis, is often overexpressed in melanoma. A number of melanoma mutations inhibit pro-apoptosis mechanisms. APAF1, a pro-apoptotic protein, is often silenced in melanoma (40%). FLIP, which inhibits death receptor-mediated apoptosis, is often overexpressed. TRAIL receptors, which induce death receptor-mediated apoptosis, can be decreased and IAPs or inhibitors of apoptosis are sometimes overexpressed in melanomas [10,11]. 

One of the first drugs to target apoptosis was an anti-sense molecule against Bcl2 called oblimersen. In phase II clinical trials of oblimersen in combination with DTIC, BCL2 expression was decreased by 42% in melanoma samples [18]. In trials with oblimersen plus DTIC versus DTIC alone there were no significant differences in overall survival between the two treatments. Since Bcl2 expression was lower in metastatic melanoma than in benign nevi and Bclxl and Mcl-1 were overexpressed in metastatic melanoma, Bcl2 may not be an optimal anti-apoptotic protein to target in melanoma [19].

In addition to avoiding apoptosis, cancers also avoid senescence, which is activated by oncogenes to halt abnormal proliferation [20]. Cancers reverse senescence by modulating genes that regulate the cell cycle such as p16^INK4a^ and p53, which increases p21 expression. Both p16 and p21 inhibit CDK to prevent Rb phosphorylation and inhibit cell cycle progression. Genes encoding Cdk4 and p16^INK4a^ are often mutated in melanoma such that inhibition of the cell cycle is abrogated. These can also serve as potential drug targets, but none have advanced to clinical trials. Other drugs for targeted melanoma therapy focus on angiogenic factors such as receptor tyrosine kinases for VEGF, PDGF, c-Kit and FLT3 [21]. 

Several issues are noteworthy for using targeted drugs. First, targeted therapies are generally effective for identified individuals with a specific mutation that the drug targets, so patients can be screened before treatment. This exemplifies the impact of personalized medicine on oncology. Second is the issue of heterogeneity within a patient’s melanoma. While a specific mutation may be determined in a sample, the targeted mutation may not be present in all of the patient’s melanoma cells. This provides a potential means for resistances and recurrences. A third issue is the continued “pressure” exerted or relieved by a targeted agent on cell signaling in the affected cancer cells. Such events modify signaling dynamics with responses that attempt to “escape” the modification, which may also lead to resistances and recurrences. These issues may complicate uses of targeted medicine in melanoma treatments.

## 2. New Approaches for Melanoma Treatment

Based on the absences of cancer treatment successes, there is little doubt that cancer treatment, especially of melanoma, requires new and innovative strategies beyond the uses of present modalities discussed above. While considerable efforts focus on development of drugs that target one or more cancer hallmarks, some alternative therapeutic approaches have been advanced that use physical methods with or without other agents that specifically target the tumor mass as a whole. The tumor is the target. One interest in tumor targeting technologies is the potential to locally affect cells and tissues within defined treatment boundaries without systemic disturbances. Two physical methods that are in practice use extreme temperature deviations such as *cryotherapy* that freezes tumors and *microwave* and *radiofrequency ablation* that thermally destroys tumors and other tissues. Both temperature-induced ablation methods primarily induce necrosis. However, these methods are not used to treat cutaneous melanomas, but have been used to treat metastatic melanomas in other organs such as liver and lung [22].

In different approaches that also focus treatments within defined boundaries, several new strategies in pre-clinical trials and/or emerging into clinical applications use non-thermal pulsed electric fields (PEFs) to eliminate cancer. Two major approaches discussed here focus on treatments that are directed to three major cancer hallmarks including avoidance of immune surveillance, evading apoptosis and sustained angiogenesis. Both approaches use electric fields in a new discipline called Bioelectrics to accomplish anti-cancer outcomes. Bioelectrics uses pulsed electric fields in the absence of drugs to control cell functions and membrane transport processes. This includes approaches that use conventional electroporation with durations of micro- and milliseconds and sub-microsecond pulses with durations of pico- and nanoseconds. One approach discussed here is called electrogene therapy (EGT) using conventional electroporation to deliver genes that locally activate one or more cancer hallmarks against melanoma. EGT is in clinical trials for treating melanoma. The other approach discusses nanosecond pulsed electric fields (nsPEFs), which extends electroporation using electric pulses to induce apoptosis and apoptosis-like features as well as to inhibit angiogenesis. NsPEFs have demonstrated effectiveness in a murine B16F10 melanoma cell culture model *in vitro* and a B16F10 ectopic murine *in vivo* model. Other studies indicate that this treatment is not limited to melanomas.

### 2.1. Nanosecond Pulse Generation and Delivery

NsPEFs utilize pulsed power technology, generating pulses that are distinctively characterized by their ultrashort duration, rapid rise-time, high power, and low energy density [23,24,25,26,27,28]. Temperature increases caused by these pulses have been measured *in vitro* and *in vivo* and the observed nsPEF effects have been shown to be non-thermal [29,30,31]. Relatively long electroporation pulses are generally considered to primarily affect plasma membranes. NsPEFs, on the other hand, affect intracellular organelles and the plasma membrane, have different pulse durations (0.1–20 milliseconds *vs*. 1–300 nanoseconds); exhibit different EF strengths (0.2–4 kV/cm *vs*. 10–350 kV/cm); have different energy densities (J/cc *vs*. mJ/cc) and different powers (500 W *vs*. 180 MW). The short duration and rapid rise time charges intracellular membranes, which do not occur with EP pulses [9,32,33,34]. 

For the definition of nsPEFs that includes intracellular effects two criteria must be met. First, pulses must have nanosecond rise times and fall times with effective rise times on the order of or less than the charging time of the plasma membrane, which is less than 100 ns. Second, pulses must have nanosecond pulse durations. For nanosecond pulse durations the first condition is automatically satisfied, so the main focus has been on pulse duration as the definitive parameter for nsPEFs.

### 2.2. Pulse Generators

Research on nanosecond pulse effects and applications for melanoma treatment require electrical pulse generators that provide well-defined high voltage pulses with fast current rise to the load. The load could be either cells in suspension or tumor tissue. The applied voltage is determined by the required threshold field for intracellular effects, which is dependent on the target, the type of cell and tissue. For single pulses, typical electric fields for inducing characteristic consistent with apoptosis range from tens to hundreds of kV/cm. The highest values for 10 ns pulses were 300 kV/cm. The threshold value also depends on pulse duration of nanosecond pulses: to achieve comparable effects with single pulses, the electric field times the pulse duration must be approximately constant. A third parameter which affects the efficiency of the treatment is the pulse number. For cells in solution it was found that the effects scale with the square root of the pulse number [35]. For tissue the effect might be proportional to the pulse number rather than its square root. In order to study intracellular electro-effects both, *in vitro* and *in vivo*, mainly pulse generator based on the Blumlein concept have been used. Details are described in a paper by Kolb *et al*. [36].

**Figure 1:**
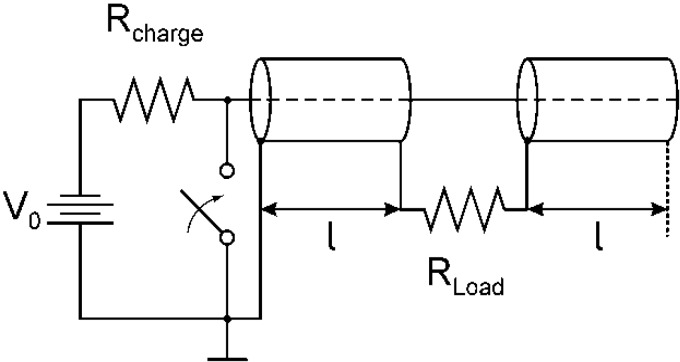
Generic circuit diagram of a Blumlein pulse generator. The two coaxial cables of identical length, l, are charged through a resistor, R_charge_, to a voltage V_0_.The two cables are discharged by closing a switch, into the load, R_L_, which for a matched system is twice the impedance of the cables.

Pulse generators based on the Blumlein concept are line-type pulsers (Figure 1). For short pulses (less than 100 ns) the pulse forming lines consist of two strip lines or two coaxial cables of equal length, **l**, with the total length determined by the desired pulse duration, τ:

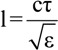
[1]
where c is the speed of light in vacuum, and e is the relative permittivity of the dielectric which separates the metallic strip lines or the conductors in a coaxial cable, respectively. For two coaxial cables of each 1 m length and a relative permittivity of 2.25 (typical for dielectrics used in cables) in a Blumlein configuration, the pulse duration, according to equation 1 is 10 ns. The Blumlein pulse generator provides a unipolar, square wave voltage pulse of the same amplitude as the voltage applied to a matched load between the two lines (matched: the load resistance has the same value as twice the stripline or coaxial cable impedance), by closing the switch at the end of one of the strip lines. In order to switch at the required voltage with a rise time of one nanosecond, a pressurized spark gap was used, operated in the self-breakdown mode. 

Such pulse generators, where spark gaps have been used as switches, have been used to apply high voltages of tens of kV to cell suspensions or tissues over electrode distances of millimeters and more, A second class of lower voltage pulse generators was designed for observations of individual cells under a microscope. Consequently, the gap distance can be reduced to the 100 µm range, and simultaneously, voltage requirements can be relaxed. Instead of typical pulsed power components, low voltage, high frequency cables can be used in the design together with fast semiconductor switches. Such types of pulse generators – micropulsers – operate at voltages of less than one kV. However, because of the small electrode gap, electric field intensities of up to 100 kV/cm are possible and can be applied to cells in suspension that are placed in the electrode gap. This type of pulse generator has been built with variations in pulse length, type of switch, type of pulse forming network, as a line type pulser, and as a hard tube pulser, in many of the ultrashort pulse studies. Circuits and designs are described in references [37,38,39,40,41]. The compactness of these pulse generators makes them standard tools for *in vitro* ultrashort pulse effect studies.

**Figure 2:**
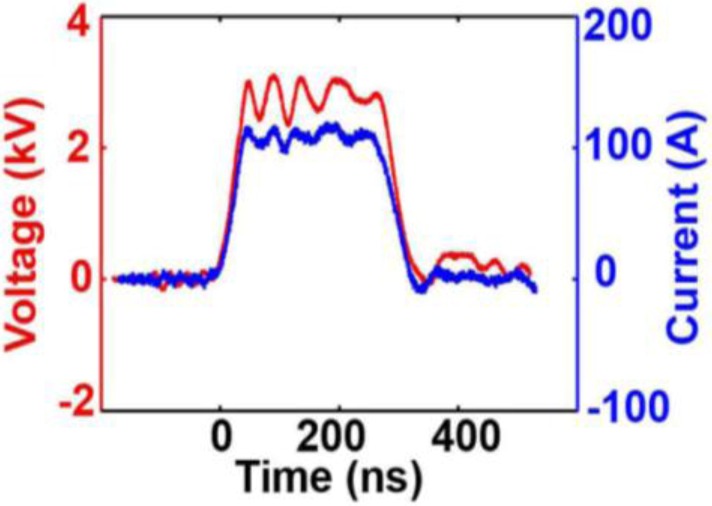
Typical voltage (red) and current (blue) pulse generated across a tumor with a 300ns pulse (reprint with permission from Elsevier [30]).

For pulses longer than 100 ns usually a pulse-forming network is used instead of cables or strip-lines. An example, such a pulse forming network was used in *in vivo* experiments described in previously [30]. The pulse-forming line which replaces the pulse forming lines shown in Figure 1, consists of 30 pairs of high voltage capacitors and 30 inductors arranged in a Blumlein configuration, and generates a 300 ns long high voltage pulse (Figure 2). The pulse was originally triggered by means of a spark gap that was later replaced by a mercury displacement relay controlled by a microcontroller.

Figure 2 (above) shows the temporal development of voltage and current used in *in vivo* melanoma studies [30]. Whereas the research team at the Frank Reidy Research Center as well as a group at BioelectromMed Corporation [42] has used 100 ns and 300 ns long pulses, respectively, a group at the University of Southern California has focused its nsPEF research on the use of even shorter pulses. In animal studies and a single human basal cell carcinoma, 20 ns long pulses were used at pulse numbers of up to 1000. The pulse generators for these studies provided pulses of typically 6.5 kV, and required relatively small electrode distances of 1.5 mm to achieve required electric fields comparable to those used at the Frank Reidy Research Center for Bioelectrics. The pulse generators used for the studies at USC were different from the ones described before. They were MOSFET based, inductive adder pulse generators with a balanced coaxial cable pulse forming network and spark-gap switch. More recent developments of pulse generators with less than 20 ns pulse duration for possible applications in skin cancer treatment have been described [43]. 

### 2.3. Pulse Delivery

**Figure 3:**
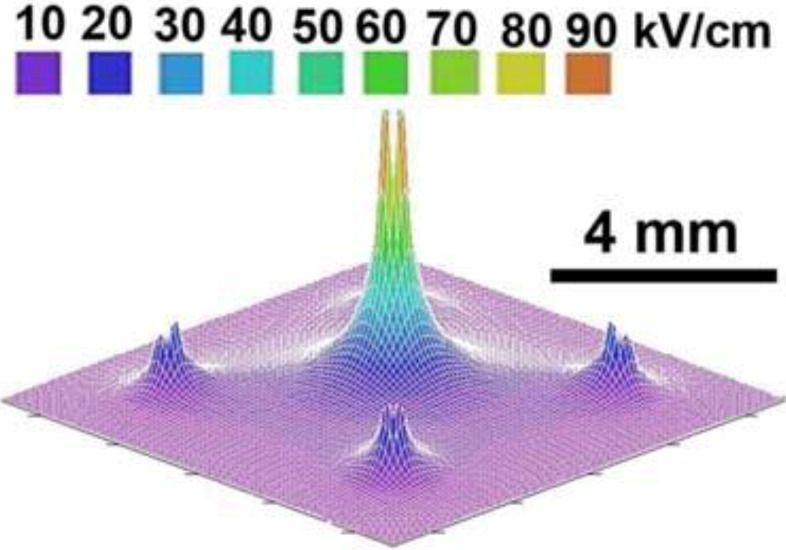
Needle array electric field pattern (reprint with permission from Elsevier [30]).

For *in vitro* studies commercially available electroporation cuvettes are often used. They have electrode gaps varying between 1 and 4 mm, and electrode areas of up to 1 cm^2^. For *in vivo* studies and medical applications generally needles, inserted into the tissue, are used as electrodes. For the *in vivo* studies performed at the Frank Reidy Research Center for Bioelectrics, the electric field was applied using two different electrode configurations [30]. The first was a 5-needle electrode array in which the needles penetrated about 2 mm into the mouse skin. The central needle was placed in the center of the melanoma to be treated and the outer four needles were outside of the boundary edges of the melanoma. This electrode array exhibits a sharply non-uniform field with field lines parallel to the surface of the skin and strongest near the center electrode (Figure 3).

**Figure 4:**
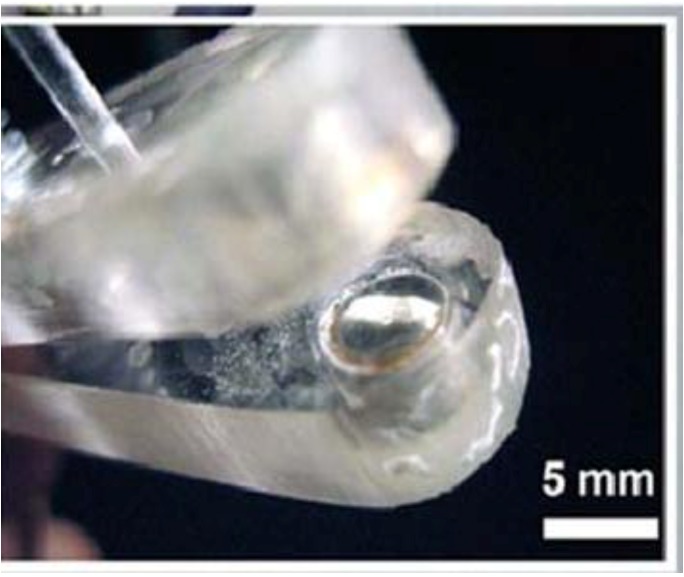
Close-up of one of the plates of parallel plate electrode showing it recessed by 0.5 mm to allow a space for addition of conductive agar gel (reprint with permission from Elsevier [30]).

Figure 3 (above) shows the electric field distribution in the five-needle pulse delivery system [30]. The second electrode configuration used involved placing the tumor between two parallel plates (Figure 4). The electric field between two parallel plates is uniform, except at the edges, so that all cells between the plates will be exposed to the same field strength. These electrodes were used when treating 48 mice by lifting a fold of skin containing the melanoma away from the mouse and placing it between the electrodes in such a way that the entire tumor is localized between the plates. This means that the field will be oriented perpendicular to the skin surface rather than parallel to it as with the needle electrodes. The distance between the plates was typically 0.5–1 mm, depending on tumor thickness. Based on our previous results with needle electrodes, we used field strength of 40 kV/cm. One difference between the two electrode types is the appearance of the skin beginning two days after treatment. A scab appears on the stratum corneum in the pulsed region and it remains for about two weeks as the stratum corneum is regenerated. Histological examination of this scab indicates that it is composed of clotted red blood cells.

Figure 4 (below) shows a close-up of one of the plates of parallel plate electrode showing it recessed by 0.5 mm to allow a space for a conductive agar gel to be placed on it [30].

### 2.4. Nanosecond Pulsed Electric Fields Effects are Different than Conventional Electroporation Effects

As indicated above, pulse power applications of ultrashort duration, rapid rise-time, high power, and low energy density distinguishes nsPEFs from classical electroporation. Modeling evidence for single cells [9,34] indicated that nsPEFs, in contrast to conventional EP, induced intracellular membrane effects. However, a more recent modeling approach suggested that electroporation pulses generate fields inside cells that are high enough to permeabilize intracellular membranes and vesicles and/or gate organelle channels [44]. This paper predicts for EP that these membrane pores allowing ionic conduction currents to expand and become larger than nsPEF-induced nanopores, but nevertheless predicts an alternative to nanosecond pulsed electric fields for intracellular manipulation. It will be important to experimentally test this in cells and tissues to discern real differences between electroporation and nsPEFs concerning intracellular effects and therapeutic relevance. What is relatively clear about nsPEFs is the concept of supra-electroporation with high density nanopores in all cell membranes [9,34]. For nsPEFs, nanopores on the order of a nanometer have been experimentally demonstrated in plasma membranes using patch clamp methodologies [45]. These nanopores exhibit ion channel-like properties, but are distinct from them. They are voltage sensitive, inwardly rectifying and affect electrolyte and water balance. The role these nsPEF-induced nanopores play, both in intracellular and plasma membranes, remain to be fully explored.

Gowreshankar and Weaver [46] modeled the effects of electric fields on tissues using an irregular-shaped, multicellular model with closely spaced cells. They compared conventional electroporation conditions (100 µs, 0.1–2 kV/cm), which is used in electrochemotherapy, with supra-electroporation conditions (300 ns, 1–80 kV/cm), which was used to eliminate B16f10 melanoma tumors *in vivo* [30]. This model shows that nanosecond pulses induced supra-electroporation with high density pore formation corresponding to an aqueous fractional area of 3.4% compared to conventional electroporation corresponding to an area of 0.02%. Further, the 300 ns pulses induced spatially homogeneous pores that included tight junctions and nuclei. In contrast, conventional electroporation included heterogeneous pore formation that excluded tight junctions and nuclear membranes. This supra-electroporation is highly likely to account for mechanisms that contribute to cell death as well as nsPEF-induced non-lethal effects. 

NsPEFs have a broad range of effects that can be above and below a threshold for cell death depending on the pulse duration, number and electric field. Much less is known about effects of pulse repetition rate. This provides new approaches to initiate intracellular signaling and affect electro-chemical kinetics from the external environment [9,23,27,32,34,47,48,49]. Applications of nsPEF activate cell signal transduction mechanisms that mobilize calcium [27,47,50,51] and activate programmed cell death (PCD) mechanisms, consistent with apoptosis *in vitro* [26,52,53,54], *ex vivo* [25] and *in vivo* [55]. In preliminary studies by Ren and Beebe, there is also evidence for induction of non-caspase-mediated cells death, such as calpain activation in E4 squamous carcinoma cells. NsPEFs activate human platelets [56], activate L-type calcium channels in chromaffin cells [57], induce action potentials in cardiomyocytes [58] and permanently eliminate B16F10 melanoma tumors in mice [30,42,55,59].

Regarding nsPEF-induced cell death, it should be considered that all signatures of apoptosis may not be observed *in vitro* because the cell membrane potential may not be maintained for extended periods of time and because of other *in vitro* conditions. This is also true *in vivo* because phosphatidylserine externalization is not easily discerned and phagocytosis may not take place rapidly in tumor masses. Furthermore, it is highly likely that multiple cell death mechanisms are activated by nsPEF treatment and their characteristic markers overlap. In general, the nsPEF treatment-specific presence of significant levels of active caspases in many cells indicate that they may have passed a “point-of-no-return”, especially in the presence of cytochrome c release and other apoptosis markers. In most instances of nsPEF-induced definitions of apoptosis, multiple methodologically unrelated assays have been used to quantify dying and dead cells as suggested in cell death literature [60,61].

## 3. Applications of NsPEFs for Melanoma Treatment

NsPEFs have been shown to eliminate murine B16F10 melanoma *in vivo* without recurrence [30,42,55,59] and in a human basal cell carcinoma [62]. Elimination of B16F10 melanomas was independent of temperature given that measured joule heating was limited to temperatures that were below the minimal temperature for hyperthermia effects [30,31]. For 300ns pulses, a threshold of greater than 20 kV/cm and an effective electric field of 40 kV/cm was needed to completely eliminate tumors for 4–5 months requiring 2–4 treatment sessions with 100 pulses per session using parallel plate electrodes [30, see Figure 4]. Histological and other evidence indicated rapid nuclear shrinkage and interruption of tumor blood supply and a decreased tumor volume that was not readily detectable by ultrasound 2–3 weeks after treatment [30,31,55]. 

### 3.1. NsPEFs Target Melanoma Cancer Hallmarks: Apoptosis Evasion

The application of nsPEFs targets at least two hallmarks of cancer: evasion of apoptosis and sustained angiogenesis, both of which are major cancer therapeutic targets. Further, effects to inhibit sustained angiogenesis implicate a third cancer hallmark- tissue invasion and metastasis. Apoptosis is an important target since it is the major form of cell death in all animals and a common target for tumorigenesis [63]. Caspase-associated induction of apoptosis-like characteristics appears to be an early nsPEF-induced mechanism that correlates with melanoma tumor demise as indicated by activation of executioner caspases, which remain active 2–8 hours after treatment in melanoma [55]. However, apoptosis may not go to completion. DNA fragments are not present as indicated by the presence of large DNA fragments and absences of ~180 bp DNA fragmentation ladders on agarose gels, a late stage apoptosis marker. However, this does not mean that apoptosis is not an effective cell recycling mechanism. It is anticipated that considerable tumor disassembly takes place early after initiation of cell death signals, providing a potential advantage for efficient tumor removal. The *in vivo* tumor masses are too large to be removed quickly by endogenous apoptosis mechanisms [55]. 

One of the largest and most complex cellular structures for degradation and removal is the genome. Morphologic and molecular evidence suggest that initial effects on DNA may be independent of apoptosis-like traits in B16F10 melanoma. Pyknosis and chromosomal condensation occurred rapidly, within the first tens of minutes after treatment [30]. Chen *et al*. [55] demonstrated the presence of histone 2AX phosphorylation (γH2AX) indicating that DNA double strand breaks were elevated one hour and occurring in 80% of cells three hours after treatment, but were essentially absent after that. Further, TUNEL positive cells, suggesting DNA damage, peaked at three hours and remain elevated six hours after treatment. However, both of these markers occurred before peak caspase activation at six hours, suggesting that major effects on DNA may not be associated with caspase activation. Other studies have indicated nsPEF-induced DNA damage *in vitro* using comet [59,64] and *ex vivo* using TUNEL [25,27,52]. However, none of these studies confirmed that these were related to apoptosis. These studies suggest that nsPEF-induced DNA damage probably occurs before apoptosis proceeds significantly and may be a direct electric field effect or more likely occurs rapidly and early as a possible secondary effect, which remains to be defined. It seems unlikely that these low energy pulses can directly induce DNA double strand breaks. Another possible mechanism for DNA damage could be generation of reactive oxygen species (ROS). However, nsPEFs are non-ionizing and may not ionize water like ionizing radiation does. However, ROS can be generated by other mechanisms that are activated by nsPEFs. Nevertheless, in contrast to treatment with ethanol or decreased temperatures, no increases in ROS were observed in B16F10 melanoma cells [54]. In yet unpublished work by Ren and Beebe, increases in ROS were not observed in E4 squamous carcinoma cells after nsPEF treatment *in vitro*. However, other cell types have not been tested. The mechanism for nsPEF-induced DNA damage requires further analysis.

### 3.2. NsPEFs Target Melanoma Cancer Hallmarks: Sustained Angiogenesis

Another nsPEF therapeutic target is tumor vasculature. There were significant macroscopic effects on tumor blood vessels within nsPEF treatment zones [30,31,55]. This appears to cause a near immediate tumor infarction, at least in some zones within the tumor [55]. Treated tumors showed increased staining for iron, a sign of hemorrhage indicating that nsPEFs caused some acute blood vessel rupture and bleeding inside the tumor [31]. More specifically, Chen *et al*. [55] demonstrated several molecular indicators for anti-angiogenesis in treated melanoma tumors. First, a decrease in vessel number supplying tumors was coincident with a decrease in tumor mass, suggesting interruption of tumor-driven angiogenesis. Second, one week after treatment there were diminished levels of the most ubiquitous pro-angiogenic factor VEGF, which is a requirement for the angiogenic switch and is a limiting factor for multistage carcinogenesis [5]. This is clearly a harbinger for the absence of revascularization and renewed tumorigenesis. Reduced levels of PD-ECGF, a well known chemotactic factor for vascular endothelial cells, provided further evidence that nsPEFs depleted the melanoma environment of needed angiogenic factors. Moreover, significant downstream VEGF effectors were reduced, including three major microvascular density markers. These included CD31, a platelet-endothelial cell adhesion molecule used as a pan-endothelial cell marker; CD34, an endothelial cell marker; and CD105, a proliferation-related endothelial cell marker. These results indicate that while melanoma tumors shrink and vessel numbers were significantly reduced, formation of new vessels was significantly inhibited. These observations indicate that nsPEFs have acute effects on tumor vasculature as well as chronic effects that discourage revascularization and tumor recurrences.

### 3.3. Mechanisms for nsPEF-Induced Apoptosis-like Effects in B16F10 Melanoma

While experiments in B16F10 tumors *in vivo* provide information about mechanisms for nsPEF-induced effects at the tumor level, *in vitro* studies provide greater flexibility to more directly determine cellular mechanisms. Experiments carried out on B16F10 melanoma cells *in vitro* also implicate apoptosis-like mechanisms [54], but not all apoptosis markers are present, nor do these cells respond to nsPEFs like other cell types such as Jurkat, HL-60, HCT116 colon carcinoma [25,52,53]. One of the most striking differences between these cells and B16F10 melanoma cells is that the melanoma cells did not release cytochrome c in response to nsPEF-induced cell death [54]. In Jurkat cells, the time courses for caspase activation and cytochrome c release were nearly coincident, suggesting that cytochrome c release and caspase-associated apoptosis were related. This indicated that the intrinsic and/or Bid-dependent extrinsic apoptosis mechanisms were operative in Jurkat cells [25]. The intrinsic pathway could be activated by intracellular stresses to the endoplasmic reticulum (ER), mitochondria, nucleus or other intracellular organelle, while the extrinsic pathway could be activated by stress to the plasma membrane that activated mitochondria mechanisms. In HCT116 colon carcinoma cells, caspase activation occurred before cytochrome c release, suggesting that the Bid-independent extrinsic pathway was activated before mitochondria-mediated apoptosis mechanisms [53]. In unpublished work, the same responses were observed in E4 squamous carcinoma cells by Ren and Beebe. This suggested that activation through the plasma membrane was the earliest response to apoptosis-like effects induced by nsPEFs in these cells. In B16F10 cells, pro-apoptotic proteins including cytochrome c, apoptosis inducing factor (AIF) and Smac/DIABLO were not released from mitochondria. This suggested that a Bid-independent extrinsic pathway was exclusively used to induce cell death and that the death signal most likely originated at the plasma membrane [53]. It should be noted that nsPEF-induced caspase-independent apoptosis mechanisms have been identified in B16F10 melanoma *in vivo* [55]. This suggests that other forms of programmed cell death are activated by nsPEFs and likely overlap with one of more apoptosis programs.

DNA damage is known to lead to programmed cell death, especially apoptosis. As discussed above, nsPEFs induce DNA double strand breaks in B16F10 melanoma tumors *in vivo* [55] and DNA damage (comet and TUNEL) in cells [59,64] and tumors [51,55]. While DNA damage precedes caspase activation in B16F10 melanoma tumors *in vivo* [55], it is possible that DNA damage contributes secondarily to apoptosis. While responses to DNA damage are extensive, many responses that lead to apoptosis act through mitochondria-mediated mechanisms.

In B16F10 melanoma tumors *in vitro*, release of pro-apoptotic factors from mitochondria were not observed. However, not all nsPEF-induced responses in B16F10 cells are fully characteristic of apoptosis, suggesting that other cell death mechanisms may be operative. For example, in response to nsPEFs, B16F10 cells did not externalize phosphatidylserine (PS), a well characterized apoptosis marker [54]. However, this appears to be dependent on the buffer used [unpublished data]. This is also surprising since nsPEFs directly induce PS externalization, irrespective of apoptosis [49,65,66]. Given that both PS and cytochrome c release require oxidation reactions, it is possible that oxidation reactions are limited by melanin and/or other radical scavengers, such as Bcl2 [67]. This is further supported by the absence of ROS in nsPEF-treated B16F10 cells [54]. Regardless of the mechanisms, B16F10 cells appear to have potent anti-apoptosis mechanisms by preventing cytochrome *c* release, a compelling survival mechanism [54]. Nevertheless, nsPEFs induce death in B16F10 melanoma cells [54] and tumors [55].

### 3.4. Advantages for nsPEFs as a Melanoma Cancer Treatment

There are a number of advantages for using nsPEFs as a means for cancer therapy as opposed to other physical methods that rely on overt necrosis for tumor cell death. These advantages include (1) multiple programmed cell death mechanisms, including apoptosis-like signatures, and anti-angiogenesis, two well known cancer hallmarks, the latter necessary for a third likely cancer hallmark, invasion and metastasis; (2) rapid death induction with minimal treatment exposures, which reduces chances for resistances and recurrences; (3) non-mitochondria-mediated programmed cell death, which can bypass many melanoma and other cancer-causing mutations; (4) effective treatment of all cells within electric fields, including rapidly growing tumor cells, slower growing host cells and cancer stem cells; and (5) minimal local and systemic side effects.

NsPEFs provide a local targeted treatment at the level of the entire tumor without systemic effects, affecting multiple molecular structures and functions in plasma membranes and intracellular organelles. All tumor cells exposed to conditions of pulse duration, number and electric field that are above the threshold for cell death are subject to programmed and other forms of cell death. The foremost targets bypass two important hallmarks of cancer causing apoptosis-like appearances and anti-angiogenesis. In its full capacity, this should lead to inhibition of invasion and metastasis, another cancer hallmark. The multi-mechanism interactions of nsPEFs with tumors are similar to using a combination of two chemotherapeutic agents and/or molecular targeted drugs that induce apoptosis-like characteristics and limit angiogenesis; both well defined sites for cancer targeted drugs. The observed decreases in vessel numbers and angiogenic factors (VEGF and PD-ECGF) prevent the possibility for re-vascularization and reduce chances for tumor cells to continue to proliferate [55]. Sustained hypoxia prevents cycles of hypoxia that have been implicated in metastasis and hypoxia induced factor (HIF) transcriptional activity that is beyond that of normal tissue [68,69]. The combination of apoptosis-like qualities and anti-angiogenesis as sites of nsPEF action makes this therapy an attractive cancer treatment [55].

Another advantage to nsPEF interactions with tumors is the rapid onset of apoptosis-like features and some level of tumor infarction. Caspase activation *in vitro* is seen within 30–45 minutes [25] and within the first hours *in vivo* [55]. This rapid caspase activation is likely to provide specific advantages by rapidly inducing cell death mechanisms. In contrast, chemotherapeutic agents, ionizing radiation, molecular targeting drugs are administered over weeks or months and often do not eliminate cancer but reduce tumor size or stabilize it. This provides a potential for mechanisms to allow tumor cells to escape therapeutic action and increases the possibility for treatment resistances and recurrences. Examples include upregulation of drug efflux transporters and tumor immune evasion in chemoresistant melanomas [70] and the chemotherapy-induced upregulation of factors like clusterin, an anti-apoptotic protein conferring resistances to several cell death agonists [71]. NsPEF-induced interruption of the tumor blood supply is also rapid, limiting blood flow to the tumor as it is being dismantled, at least in part by apoptosis-like mechanisms. NsPEF treatment has rapid therapeutic onset, which should reduce the potential for resistances and recurrences as all tumor cell are affected by conditions above the threshold for cell death. 

Many mutations that lead to cancers occur in mitochondria-mediated mechanisms, most likely because there are more regulatory sites through intrinsic and Bid-dependent extrinsic pathways than in mitochondria-independent apoptosis pathways. Consequently, many chemotherapeutic agents and ionizing radiation have significant effects on mitochondria-dependent apoptotic mechanisms [72]. NsPEFs have both mitochondria-dependent and -independent sites of action that appear to be cell type dependent. In melanoma, the exclusive recruitment of mitochondria-independent extrinsic mechanisms provides an alternative mechanism to many cancer therapeutic treatments that act on mitochondria-dependent pathways. A simple, bistable rate-equation based model of apoptosis pathways predicted that the extrinsic caspase-8 mechanism was more sensitive than the mitochondrial intrinsic pathway for electric pulse induced cell apoptosis [73], which is in keeping with results from B16F10 melanoma [54] as well as HCT116 colon carcinoma [53]. Thus, by favoring the extrinsic apoptosis pathway, nsPEFs may bypass many cancer causing mutations in mitochondria-mediated apoptosis mechanisms, which are often involved in resistances and recurrences. 

Another potential advantage of nsPEF as a cancer therapy is related to considerations for cell type specificity. Chemotherapeutic drugs and ionizing radiation primarily affect rapidly dividing cells. NsPEFs have some cell type specificity, but it may not have therapeutic relevance. Cultured cells that grow attached as opposed to cells in suspension require longer pulse durations, greater numbers of pulses and/or higher electric fields to elicit cell responses [64], including cell death [25,53,54]. In contrast to conventional electroporation, which affects larger cells more readily than smaller ones, cell size did not matter for plasma membrane permeabilization with nsPEFs [74]. However, there is no evidence that nsPEFs preferentially affect only rapidly proliferating cells. S phase synchronized cells under limiting nsPEF conditions exhibited greater membrane integrity and maintained cytoskeletal structure but did not differ in survival compared to unsynchronized cells [75]. Thus, within a heterogeneous tumor mass, nsPEF therapy is expected to induce cell death in rapidly proliferating tumor cells as well as slower proliferating host cells that are collaborating with tumor cells regardless of their size. This suggests an alternative to almost all therapeutic regiments that predominantly target rapid proliferating cells. Melanoma tumors also can contain cancer stem cells or other slower cycling cells, which possess characteristics common to normal stem cells, including self renewal capacity, high tumorigenicity and potential to differentiate into multiple cell types [76,77,78,79]. Cancer stem cells or other slower cycling cells may be more prevalent in tumors than initially considered as demonstrated with melanomas from 12 different patients [80]. Herlyn and colleagues have suggested an alternative to the unidirectional stem cell model in melanoma proposing a dynamic temporarily distinct subpopulation of slow cycling melanoma cells that are responsible for tumor maintenance [79] The existence of these slow cycling cells is clinically relevant because they would be resistant to most therapeutic regimens [79], but probably not to nsPEF therapy. Cancer stem cells or slow cycling cells have been reported to be responsible for recurrences after chemotherapy and ionizing radiation therapy through multiple mechanisms. One of these mechanisms is to minimize therapy-induced DNA damage that is produced by free radical scavengers to minimize the effects of reactive oxygen species (ROS). Cancer stem cells had significantly lower levels of ROS and enhanced ROS defenses compared to non-tumorigenic cells [81]. NsPEFs are non-ionizing and they do not appear to induce cell death by generating measurable ROS in B16F10 melanoma cells [54]. Thus, this mechanism would not provide survival advantages to cancer stem cells exposed to nsPEFs. Another mechanism that may be responsible for resistances and recurrences with conventional treatments is to preferentially activate DNA damage checkpoint response and increase in DNA repair capacity [82]. NsPEFs do cause DNA damage in B16F10 melanoma tumors [55]. However, DNA damage may not be a major cause of cell death in these tumors [55]. Furthermore, DNA damage induces apoptosis through release of pro-apoptotic factors from mitochondria [83,84,85] and nsPEFs induce melanoma cell death in the absence of release of pro-apoptotic factors [54]. Thus, minimizing DNA damage and enhancing repair would not provide survival advantages to cancer stem cells or slow cycling cells exposed to nsPEFs.

An important benefit to local treatment with nsPEFs is an absence of side effects and toxicities, which are common with nearly all systemic treatments, especially chemotherapy and ionizing radiation. In studies with mice, nsPEFs have minimal and resolvable effects on skin. With parallel plate electrodes that eliminated B16F10 melanoma, the stratum corneum showed signs of necrosis and hemorrhage with accompanying superficial erosion of the epidermis [30]. However, these characteristics appeared two days after treatment, differentiating the effect from burn or heat related injuries, which occur immediately. With a four plus one needle array electrode, nsPEFs caused some edema and bleeding, but the damage was resolved within a week [31]. Small scabs formed but were resolved within two weeks and did not leave a scar. However, mice do not readily scar. In an unpublished clinical study observing effects of nsPEFs on human skin, treatments with two parallel needle electrodes caused some irritation, redness and itching at insertion/treatment sites, which were readily relieved by anti-histamines, local anti-inflammatory ointment and protection from scratching. The treatment caused no permanent scars or discoloration of skin regardless of pigmentation. While there was some pain and discomfort with applications of nsPEFs without anesthesia, they were eliminated when a local anesthetic was injected at treatment sites. In addition, nsPEF caused no muscle contractions like those observed with conventional electroporation and irreversible electroporation. In addition, studies monitoring general reactions to nsPEFs with parallel plate electrodes, mice had slightly higher heart rates and respiratory rates, but body temperature and systolic blood pressure did not change significantly [31]. Thus, as tested so far, applications of nsPEFs are generally safe, non-toxic and without scarring or other permanent effects on skin in mice and humans. While nsPEF treatments can now be used for surface tumors using needle or plate electrodes, applications to internal tumors will likely be possible as catheter electrodes are developed for laparoscopic surgeries. For all nsPEF treatments, multi-needle electrode systems with adjustable field orientations would likely enhance apoptosis in the context of pulsed voltage-induced inactivation of tumor cells [73].

## 4. Applications of Conventional Electroporation for Gene Delivery and Melanoma Treatment

With potential problems inherent in viral vector-mediated gene delivery, non-viral gene therapy has become more attractive for treatments of cancer. Several non-viral approaches have been investigated including the use of plasmid DNA or protein-DNA complexes placed in contact with cells to be transfected or delivered by microinjection, liposomes, calcium phosphate precipitation, gene gun, ultrasound cavitation (sonoporation), hydrodynamic and electroporation. Conventional electroporation, which is the focus of bioelectric approaches to cancer treatment, has been used for over a quarter of a century to deliver genes to cells [86,87]. While the delivery of genes and DNA to cells by conventional electroporation has played a major role in molecular cell biology with transformation of bacteria and transfection of cells in basic science studies, the delivery of drugs or genes to tumors and other tissues has therapeutic application in the practice of medicine. One of the early efforts to deliver molecules to tissues for therapeutic purposes was to deliver poorly permeable chemotherapeutic agents such as bleomycin to induce cell death and tumor regression in animals [87,88,89,90,91,92,93,94,95,96]. A number of studies were carried out using bleomycin or cisplatin delivery in mice showing both safety and efficacy with various electroporation conditions and delivery devices [93,94,95]. Further, electroporation methodologies for drug and gene delivery did not significantly change gene expression profiles in malignant melanoma cells, especially not tumor suppressor genes, oncogenes of cell cycle regulation or genes involved in the stability of DNA. Only seven out of 2698 genes exhibited changes in expression, including a stress related protein, a gene involved in chromatin assembly and down regulation of genes involved in protein synthesis [96]. It was also shown that only minor histological changes occurred in electroporated muscle with no changes in gene profile for cell death, inflammation and muscle regeneration [97]. In another study only two out of 140 genes changed expression levels and none involved in stress or toxic responses [98]. Thus electroporation is safe and does not promote tumorigenesis.

Successes in drug delivery to tumor tissues provided the basis for gene deliver to animal cells and tissues *in vivo*. Early gene therapy methods used viral-mediated gene transfer, but a number of safety concerns mounted including potential for toxicity, immune and inflammatory responses and the possibility that the virus may recovery its ability to cause disease. Further, incorporation of the viral vector into a site of a functional protein, such as a tumor suppressor gene, could lead to tumorogenesis. However, the onus of non-viral gene therapy would be to achieve sufficient levels of gene expression to reach therapeutic efficacy. Non-viral methods included systemic liposome-mediated gene delivery [99,100], or injection of calcium phosphate precipitated DNA vector into liver, spleen [101], into the peritoneal cavity with expression in liver and spleen [102] or muscle injection [103].

The first reported attempt to deliver genes using electric pulses into newborn mice skin used a plasmid with a neomycin-resistance gene controlled by SV40 early promoter and including a gene for the SV40 T-antigen to transform cells and a plasmid containing the E1A region of Adenovirus 2, which could immortalize cells [104]. Neomycin resistant primary fibroblasts were cultured *in vitro* from treated skin, restriction fragments from the SV40 promoter and the E1A gene of adenovirus were identified in total DNA samples from clones on Southern blots and expression of the SV40 large T-antigen was found on Northern blots. These and other results suggested that the genes were integrated into the chromosomes. While gene delivery was accomplished *in vivo*, gene expression was carried out *in vitro*. 

In another approach, C6 glioma cells were inoculated into the right striatum of rats to form brain tumors. Ten days later the tumors were electroporated followed by injection of a bacterial *LacZ* gene specifically into the brain circulation. The technique resulted in efficient and controlled gene transfer to the brain tumor. A major limitation of this approach was the potential for non-specific delivery of the plasmid to unwanted tissues. However, β-galactosidase activity was evident in tumors 3 weeks after electroporation treatment, but surprisingly not in other brain regions or other organs [105].

In keeping with the introduction of gene expression vectors directly into organs, electroporation of rat liver with a plasmid that expressed luciferase or β-galactosidase was the first example of electroporation-mediated gene delivery and expression directly into an organ *in vivo* [106]. Optimal conditions for electroporation established dose-dependent luciferase expression kinetics, peaking on day two and maintaining significant expression for three weeks. β-Galactosidase expression was also demonstrated in isolated hepatocytes by flow cytometry. Histological examination indicated the absence of tissue damage and that expression was broadly and randomly distributed within the electroporated tissue. This study demonstrated that efficient gene transfer and expression could be achieved in sufficient numbers of cells with an electric field to be of therapeutic interest.

While initial interest in gene therapy focused on correction of single gene defects in hereditary diseases, gene therapy for cancer treatment has received the most attention for therapeutic application in clinical trials. Following the study by Heller *et al*. [106], a number of other studies confirmed the simplicity, convenience, efficacy and safety of *in vivo* gene delivery by electroporation in wide range of tissues in several different species demonstrating the potential for therapeutic applications. Muramatsu *et al*. [107] electroporated and successfully expressed a LacZ reporter gene driven by the testes specific mouse-protamine 1 promoter in spematogenic-like cells in mouse testis. This same group also showed that electroporation-mediated delivery of a lacZ reporter gene driven by the chicken actin promoter was superior to microparticle bombardment and lipofection for gene delivery to somatic cells in early chicken embryos in ovo [108]. Rols *et al*. [109] demonstrated intratumoral delivery of both the β-galactosides protein and a reporter plasmid carrying the gene in murine B16 metastatic melanoma tumors. Other studies established electroporation-mediated delivery of a green fluorescent protein reporter plasmid in rat liver [110], a plasmid for IL-5 expression in mouse muscle [111] and long term (9 months), high level expression of a reporter plasmid in muscle [112].

The most common animal/tumor model that led to beliefs in therapeutic possibilities in tumors, as well as in muscle or skin, was the C57Bl/6 mouse harboring B16F10 melanoma tumors. Gene therapy for cancer has focused on several basic strategies including immune potentiation, suicide gene therapy, restoration of tumor suppressor genes and/or inhibition of oncogenes, anti-angiogenic genes, genes encoding toxins or siRNAs to knockdown proteins important for survival and growth [113,114]. Although the roles played by these anti-tumor expression products are often multifaceted, complex and not fully defined, considering the hallmarks of cancer [5,6], electrogene therapy has aimed to overcome essentially all of them with the exception of invasion and metastasis. However, since genes responsible for metastasis have not been specifically identified, the inhibition of sustained angiogenesis indirectly addresses this category. While these hallmarks have been addressed by electrogene therapy, the most attempted and successful strategy has been the evasion of immune surveillance. Nevertheless, further consideration for expression of many of these gene products is prudent. 

### 4.1. Gene Therapy to Prevent Apoptosis Evasion in Melanomas

In efforts to overcome apoptosis evasion in melanomas, several different electrogene therapeutic approaches have been investigated. In one effort, pro-apoptotic genes apoptin and E4orf4 were delivered by electroporation into B16F10 tumors. Apoptin, a protein encoded by chicken anemia virus, induces cell death by apoptosis [115]. It induces G2/M cell cycle arrest and activation of caspases through an intrinsic mitochondria mechanism [116,117], in some but not all cell types. E4orf4, the protein encoded by open reading frame 4 in the E4 region of adenovirus, promotes cell death by p53-independent apoptosis and is specific for transformed cells. Apoptosis induction by E4orf4 requires binding to protein phosphatase 2A and involves downregulation of MYC, a multifunctional transcription factor involved in cell growth, differentiation, genomic stability, cell motility, cell adhesion and apoptosis [118]. These could be effective suicide genes, but unfortunately, gene transfer and/or expression were too low and tumor growth inhibition was not a permanent therapeutic effect.

A more successful series of studies utilized another viral protein, HIV-1 Vpr (accessory protein R) in B16F10 tumors. Vpr regulates a number of cell functions including cell cycle arrest at G2/M and subsequent p53-independent apoptosis. B16F10 cells transfected with Vpr were less effective in colonizing lung tissue than non-Vpr-B16F10 cells, inhibited *in vitro* growth and preferentially affected rapidly proliferating cells and resulted in tumor growth attenuation and complete regression in some tumors [119,120,121,122]. Further support for electrogene delivery of Vpr as an anti-cancer agent comes from the demonstration of *in vitro* growth inhibition with peptides from the carboxy-terminal third of Vpr [123], which encodes part of the third alpha helix [124] and contains part of the sequence with the greatest effects on viability [125].

### 4.2. Gene Therapy to Avoid Sustained Angiogenesis in Melanomas

Survivin is a member of the inhibitor of apoptosis (IAP) family, which functions to inhibit the activity and the activation of caspase proteases, affecting both the extrinsic and intrinsic apoptosis pathways [126]. While survivin is an anti-apoptotic protein, it plays a particularly important role in endothelial cells, where its expression is increased by VEGF, inhibiting apoptosis during vasculogenesis and angiogenesis [127,128]. Further, it plays an important role in cell cycle regulation, where Cdc2 phosphorylation of survivin on Thr 34 stabilizes an anti-apoptotic complex during metaphase to allow cell cycle traverse, providing cytoprotection to proliferating cancer cells [129]. In its dual role in apoptosis and proliferation, bridging cell death and survival, its prominent role in angiogenesis and the consequences of overexpression in cancer, including melanoma [130], make it an excellent target for cancer therapy. Further, since it is mostly absent in differentiated cells and overexpressed in tumors [126], and antibodies to survivin have been found in sera from some cancer patients [131], it is considered a tumor-associated antigen and is an attractive target for T cell-based immune strategies against cancers. Using in silico epitope prediction algorithms and binding to HMC class I molecules, Lladser and co-workers [132] delivered a CD8+ T-cell epitope of survivin_20–28_ by intradermal electroporation. Expression from the survivin coding plasmid produced CD8+ cytotoxic T cell response with cross reactivity to the mouse survivin_20–28_ as determined by INFγ staining. In addition, intradermal delivery of a plasmid encoding the full length survivin suppressed angiogenesis and provided protection against a challenge from aggressive B16 melanoma. These results provide the motivation for further analysis of intradermal electroporation as a means of survivin or other DNA vaccination.

Another electrogene therapeutic strategy for melanoma is designed to inhibit sustained angiogenesis. This approach was to electro-transfer a plasmid expressing endostatin. Endostatin, a 20 kDa C-terminal fragment of collagen XVIII, specifically inhibits endothelial proliferation and inhibits tumor growth and angiogenesis. It is an inhibitor of Wnt signaling, which promotes β-catenin degradation that prevents transcription of a number of genes, including cyclin D. This is consistent with endostatin inhibition of cyclin D1 promoter activity, which causes G1 arrest in endothelial cells, reinforcing the idea that catenin is a target for endostatin [133]. Thus, Wnt signaling plays an important regulatory role in the vasculature and appears to be critical to angiogenesis [134]. Considering this, it was shown that electrotransfer of endostatin into muscle tissue resulted in reduced numbers of B16F10 tumor in the lung, demonstrating the electrogene transfer can be successfully used to deliver anti-angiogenic genes and prevent neoplasia in tissues [135].

Another anti-angiogenesis approach was to deliver vasostatin, the N-terminal domain of calreticulin inclusive of amino acids 1–180 [136]. Vasostatin is a potent angiogenesis inhibitor. It selectively inhibits basic fibroblast growth factor (bFGF)-induced endothelial cell proliferation *in vitro* and bFGF-induced angiogenesis and neovascularization *in vivo* [137]. Using the B16F10 melanoma model, a vasostatin plasmid was electroporated into hind limb tibial muscles and cyclophosphamide, a pro-drug, converted to an active chemotherapeutic DNA alkylating agent in the liver, was injected intraperitoneally over a period of days. The combination of the two therapies was better than either one alone. There was both a significant inhibition of tumor growth and an extended survival of treated mice. About 10% of mice treated with vasostatin and cyclophosphamide survived for 3 months compared to no survival at 53 days with vasostatin alone. The authors suggested alternative strategies for combining both treatments could improve therapeutic reliability.

Continuing to use the concept of electrogene therapy for anti-angiogenesis, Chan *et al*. [138] co-delivered angiostatin and endostatin, both shown to inhibit angiogenesis, in combination with three melanoma-associated antigens. These included tyrosinase-related protein 2 (TRP2), which had been shown to be expressed in a variety of cancers including melanoma and had shown clinical tumor regression with TRP2-specific T-cells [139]; gp100, a melanosomal matrix protein whose expression is closely correlated with cellular melanin content, a frequent melanoma tumor antigen recognized by cytotoxic T lymphocytes and expressed in patients with metastatic melanoma [140,141]; and PADRE (AKXVAAWTLKAAA, where X is cyclohexylalanine), a linear carbohydrate-peptide construct based on the 13 amino acid non-natural pan class II epitope. The melanoma vaccination together with expression of angiostatin and endostatin resulted in 57% tumor-free survival for over 90 days after challenge [138].

Another strategy to eliminate melanoma is to downregulate signal transducer and activator of transcription 3 (STAT3). STAT3 is a cytoplasmic transcription factor that is activated by cytokine or growth factor binding to their respective receptors. It is overexpressed constitutively in cancers of diverse origins. It is involved in transcription of a multiplicity of genes that cover most of the cancer hallmarks including controlling proliferation, differentiation, apoptosis, angiogenesis, metastasis, immune evasion and tumor survival [13,142]. The importance of STAT3 in oncogenesis makes it an excellent target for cancer therapy. To prevent the function of this protein, a dominant-negative STAT3 construct was used in an electrogene therapy strategy that transferred dnSTAT3 into B16F10 tumors [143]. The growth of STAT3 treated tumors was significantly inhibited in a majority of mice. TUNEL staining, as an apoptosis marker, indicated that expression of dnSTAT3 resulted in apoptosis in greater than 50% of treated tumor cells and as great as 90% in two mice. Further, there were more cells that were apoptotic then were transfected, suggesting a bystander effect. This bystander effect could be explained, at least in part, by the release of soluble factors in B16F10 cells upon inhibition of STAT3, such as TRAIL, which can induce apoptosis [144]. As might be expected, gene knockout or siRNA effects targeting STAT3 have multiple effects on melanoma, including inhibition of HIF1ά expression, which turns off many genes involved in vasculogenesis and angiogenesis; inhibition of endothelial cell migration and vessel formation; and initiation of pro-inflammatory cytokine and chemokine production, which activates innate immune cells and initiates anti-tumor immune responses. Increasing or overexpressing STAT3 has also been shown in melanoma cells to upregulate the pro-survival proteins MCL1 and BCL2; induce VEGF, the most potent angiogenic factor; downregulate p53, which can induce cell cycle arrest and apoptosis; and activate matrix metalloproteinases, promoting melanoma metastatic capacity [13]. Many of these same STAT3 effects were observed in studies using human cells in nude mice, human melanoma cell lines and human tissue specimens. In the mouse model, brain metastasis was increased when STAT3 was constitutively expressed and decreased when dnSTAT3 was overexpressed [145]. It was also shown that STAT3 activity in melanoma cells affects recruitment of diverse immune effectors and it can be manipulated to activate the effector phase of innate immune responses [146].

### 4.3. Gene Therapy to Avoid Evasion of Immune Surveillance in Melanoma

One of the most frequently applied cancer treatments with electrogene delivery is directed towards the cancer hallmark, evasion of immune surveillance. There is increased recognition that the relationship between tumors and host immunity plays critical roles throughout the diverse stages of tumorogenesis and that understanding and manipulating these interventions has important therapeutic benefits for controlling melanoma and other cancers [147]. One of the most investigated and successful electrogene delivery strategies is intratumoral delivery of IL-12 followed by electroporation. IL-12 is an interleukin that activates T-cells, stimulating their growth and function. It is mainly expressed by dendritic cells. IL-12 regulates innate and adaptive immune responses to pathogens and tumors and produces protective immunity by promoting Th1 differentiation of CD4+ cells, engaging CTL into tumors through the induction of various cytokine mechanisms and stimulating macrophage and NK cell cytotoxicity. It also starts anti-angiogenesis mechanisms by directly activating INFγ and inhibiting VEGF and matrix metalloproteinase 2/9 [148]. T-cells and natural killer (NK) cells respond to IL-12 by the production of tumor necrosis factor-alpha (TNF-α) and interferon-gamma (IFN-γ) and attenuation of IL-4-mediated repression of IFN-γ, providing immunoregulatory function and anti-tumor activity [149]. In addition, IL-12 has recently been shown to have direct anti-tumor activity on murine B16 melanoma cells expressing functional IL-12 receptors [150]. However, systemic administration of recombinant IL-12 produced significant toxicities. Nevertheless, antitumor activities have been demonstrated by transferring IL-12-expressing tumor cells or plasmids expressing IL-12. Electrogenetherapy with IL-12 has been investigated with a number of electroporation conditions showing tumor regression and prolongation of survival.

Lucas and Heller [151] examined a wide range of electroporation conditions in mouse skeletal muscle delivering a plasmid encoding IL-12. Parameters included millisecond pulses with low voltage (40–200 V/cm) and microsecond pulses with high voltage (750–1500 V/cm). Of the conditions tested, low-voltage, millisecond pulses resulted in higher, prolonged expression of plasmid DNA than high-voltage, microsecond pulses. Using 20 milliseconds, 100 V/cm pulses, IL-12 and a downstream effector IFNγ were both elevated for as long as 21days. This in-depth study provided evidence and protocols for electroporation-mediated immune-modulation delivering IL-12 to skeletal muscle. Heller and colleagues [152] also demonstrated that IL-12 delivery to skin by electroporation was about 10 times better than injection of the plasmid without electroporation. Lohr and co-workers [153] showed that intratumoral injection of IL-12 or IL-2 with electroporation gave similar levels of expression as intratumor delivery of IL-12 by an adenovirus expressing IL-12; however, serum levels were higher and toxicities were present with adenovirus delivery. Kishida *et al*. [154] delivered IL-12 and IL-18 using an Epstein-Barr-based plasmid replicating vector containing EBV EBNA1 gene, which exhibits several functions including nuclear plasmid transfer, plasmid binding to the nuclear matrix and up-regulation of gene expression. This EBV-based vector expressed 20-times higher luciferase levels than the conventional plasmid, while IL-12, IL-18 and IFNγ serum levels with the EBV-based plasmid were only about 1.4, 3.3 and 3.0 times higher, respectively. IL-12 gene transfection resulted in significant tumor growth suppression and the therapeutic effects were enhanced by co-transfection with IL-12 and IL-18. When tumors were treated repetitively on days 0, 2, 10 and 12, results were significantly better than treatment on days 0 and 2 and 70% of mice survived at least 40 days. NK and CTL activities were significantly higher with co-transfection of the two cytokines. The high expression levels and serum levels of IL-12, IL-18 and IFNγ may be due to the use of the replicating EBV-based plasmid and/or to the intense pulsing conditions. No toxicities were reported. Lucas *et al*. [155] compared IL-12 electroporation delivery into tumors and muscle in the B16F10 melanoma model. Tumor deliver was clearly better than muscle delivery for effects on tumors. While serum levels of IL-12 and IFNγ after muscle delivery were present, there was no tumor regression. Tumor delivery resulted in anti-angiogenesis effects observed by decreases in blood vessel numbers and decreases in levels of the micro-vessel density marker CD31. Forty seven percent of tumor bearing mice survived for at least 100 days, considered a cure. Tumor delivery resulted in influx of CD4+ and CD8+ cells into tumors and 70% of tumor-bearing mice were resistant to challenge. Further, administration of IL-12 by electroporation did not result in tumor regression in nude mice with either delivery to tumors or muscle. This supports a role for T-cells in tumor regression in response to IL-12.

Kishida *et al*. [156] combined electrochemotherapy using bleomycin and electrogene therapy using IL-12 with intratumoral delivery and intravenous inoculation with B16F10 tumors. Serum and tumor levels of IL-12 were not different when IL-12 was transfected alone or in the presence of bleomycin. Nearly 38% of mice that received both agents in tumors showed complete remission and were resistant to challenge. With intravenous injection of melanoma, mice that received IL-12 and bleomycin showed significantly longer mean survival time and cytotoxicity activities of NK and CTL. In contrast, mice that received IL-12 alone exhibited similar numbers of metastatic foci to the co-treated mice, but only the combination therapy significantly prolonged mean survival time of tumor bearing mice and only partially elevated NK activity. The study indicated that electrochemogene therapy elicited marked suppression of treated mice and innate and adaptive anti-melanoma immunity. IL-12 alone and IL-12 plus bleomycin suppressed bystander metabolic lesions.

Lucas and Heller [157] studied effects of IL-12 electrogene therapy on primary and secondary tumors in the B16F10 murine melanoma model. With three treatments of intratumoral IL-12 delivery, 80% of treated mice with B16F10 tumors were tumor free for greater than 100 days, suggesting a cure. The cured mice were resistant to challenge with a second injection of B16F10 tumor cells. In another approach, two B16F10 tumors were injected. When the first tumor was treated, a second injection of B16F10 tumor cells was established on the opposite flank. Only 43.8% of mice treated with two or three electrogene treatments developed the second distant tumor, compared to 87% of age-matched control mice injected for the first time. In another approach, B16F10 cells were injected intravenously and tumor development in the lungs were analyzed after intramuscular IL-12 electrogene therapy. Only 37.5% of mice electrogene treated with IL-12 in muscle developed nodules in the lung, while 87.5% of mice not treated developed nodules. These studies established IL-12 as an effective therapy for primary and distant tumors as well as metastatic B16F10 tumors in mice. 

A thorough toxicity study with electrogene transfer of IL-12 was required in mice before this therapeutic modality could be introduced into clinical trials [158]. For mice receiving IL-12 encoded constructs, no significant toxicities were observed. Only minor histopathologies were found and some inflammation in the kidney. The animals were healthy, indicating a diminished disease burden. 

The first phase I electrogene clinical trial was carried out by delivery of IL-12 into metastatic melanoma tumors in patients [159]. The study was designed to determine safety and tolerability and the correct dose of this type of treatment as well as its effectiveness in treating melanoma. While clinical trials with electroporation had been carried out with drug delivery, the combination of plasmid injection and electroporation was tried here in humans for the first time. Toxicity profiles indicated that electrogene delivery was safe, well tolerated with minimal toxicity and only transient pain sensed during delivery of electric pulses. The maximum tolerated (dose 5.8mg/treatment), was the highest IL-12 plasmid dose tested. There were both local and systemic responses observed. Forty two percent (eight out of 19) of patients showed disease stabilization and 10% (two out of 19) with non-electroporated lesions and no other treatment therapy showed complete regression of all metastases. Post treatment biopsies indicated plasmid dose-dependent responses. Levels of IL-12 and IFNγ increased as much as 18-fold and 7- to 60-fold, respectively, in tumors over median baseline measurement for the entire study group. No increases in IL-12 or IFNγ were observed in serum samples. The successful outcomes of this trial led to phase II trials, which are in progress.

In a phase 1 clinical trial of electrogene delivery of IL-2 to patients with malignant melanoma, with maximum tolerated dose 5.0 mg/tumor injection site, responses were observed in treated and untreated lesions, indicating decreased tumor size and local and systemic activity. No serious adverse events were reported other than Grade 1 due to drug injection and/or the electroporation procedure [160]. These clinical trials with IL-12 and IL-2 treatment for metastatic melanoma demonstrate safety, efficacy and systemic immune responses, validating electrogene delivery as an important new addition to cancer treatment modalities. Other animal studies have indicated that this method can be used alone or in combination with other therapies, including electrochemotherapy.

In contrast to electrogene delivery and expression of IL-12, similar studies in B16F10 tumors in mice with IL-2 or GM-CSF (granulocyte macrophage colony stimulating factor), a cytokine secreted by macrophages, T-cells and others that functions as a white blood cell growth factor, were not sufficiently effective to provide a survival benefit [161]. In spite of good luciferase expression, expression of GM-CSF in electrogene treated tumors was apparently too transient to be effective to significantly slow tumor growth. However, electrochemotherapy treatment with bleomycin, which resulted in short term, complete regression but no resistance to challenge, followed by electrogene treatment with IL-2 or pretreatment with GM-CSF caused long term immunity to recurrences and resistance to challenge in 25% of treated mice. However, while the GM-CSF plasmid was delivered directly to the tumor, the IL-2 electrogene treatment needed to be peritumoral and not intratumoral. Apparently, IL-2 expression in healthy tissue surrounding the tumor was sufficient to generate a survival advantage as well as long-term antitumor immunity in B16 mice pretreated with electrochemotherapy. Given successes in phase I clinical trials with IL-2, the limited success with IL-2 mouse melanoma suggests that higher plasmid levels for electrogene treatments may find better success with IL-2 and GM-CSF without electrochemotherapy. This is further supported by previous studies with irradiated B16 melanoma cells transformed with retroviruses individually expressing one of 10 different gene products, founding that GM-CSF was the most potent molecule tested [162]. While irradiation alone was ineffective, GM-CSF expressing irradiated cells exhibited long lasting and specific anti-tumor immunity, requiring both CD4+ and CD8+ cells.

Interferon alpha (IFNά) was the first cytokine to show efficacy in cancer patients including those with melanoma [163]. IFNά binds to its receptor and activates the JAK-STAT signaling complex resulting in activation of p38 mitogen-activated protein kinase (MAP kinase) and phosphatidylinositol 3-kinase (PI3K) signaling pathway [164] to promote the differentiation and activity of host immune cells generating long-lasting antitumor responses [165]. To determine if IFNά was an effective agent for electrogene therapy, Heller *et al*. [166] delivered IFNά to mice with B16 melanoma tumors directly into tumors or into the gastrocnemius muscle. Intratumoral delivery, but not intramuscular delivery slowed growth and induced complete and long term regression in mice. Seventy percent of mice were tumor free for at least 75 days with the highest plasmid dose tested.

IL-15 has also been used as an immune stimulant in the treatment of melanoma in mice. IL-15 is structurally similar to IL-2 and they share many biological activities. One of the most important IL-15 functions is to promote memory CD8+ T cell and natural killer cell survival, both of which are crucial for tumor immune surveillance [167,168]. In IL-15 receptor knockout mice, natural killer cells did not survive [169]. To determine if IL-15 would support survival in mice with B16F10 melanoma, Ugen *et al*. [170] tested delivery of an IL-15-plasmid by electrogene transfer. Results indicated that 37.5% of mice receiving IL-15 by electroporation survived with complete B16F10 tumor regression. Like studies with electrogene transfer of other cytokines, this emphasizes the potential clinical use of plasmid treatment of malignant tumors by electrogene delivery. 

### 4.4. RNA Interference and Electrogene Therapy for Melanoma

In essentially all of the examples of electrogene therapy discussed so far, the plasmids used increase the expression of genes that in one way or another inhibit or eradicate melanoma tumors. A more recent strategy is to interfere with expression of genes that exemplify cancer hallmarks and promote tumorogenesis. While this can be done by overexpressing dominant negative cDNA plasmid mutants, such as with dnSTAT3 as discussed earlier, another methodology is therapeutic applications of RNA interference (RNAi) or microRNAs. This tactic makes use of the discovery of intrinsic microRNAs that impede the expression of genes by post-transcriptional gene silencing mechanisms, triggered by small interfering double-stranded RNA (siRNA) with degradation of mRNA homologous in sequence to the siRNA [171,172,173]. This can be done by the delivery of mature siRNA molecules or as short hairpin RNAs (shRNAs) using plasmids. This introduces a new dimension into nucleic acid based therapeutics for gene therapy. Takahashi *et al*. [174,175] demonstrated that RNAi was effective to suppress luciferase expression in B16-BL6 melanoma cells stably expressing both firefly and sea pansy luciferase after intratumoral injection and electroporation in mouse footpads using either siRNA or siRNA-expression plasmids. Luciferase expression was decreased by about 60% of control values 24 hour after treatment. Golzio and colleagues [176,177] also demonstrated feasibility of silencing enhanced green fluorescent protein (EGFP) with electrically mediated delivery of siRNA in mice bearing stably expressed EGFP B16F10 melanoma tumors. They demonstrated gene silencing that lasted 2–4 days after a single treatment with electric field-mediated delivery of siRNA using fluorescent imaging in mice as well as conformation of decreased EGFP by quantitative PCR.

Beyond the siRNA-mediated knockdown of reporter genes, the use of shRNA using electrogene therapy by intratumoral injection of RNA expressing plasmids targeting β-catenin or hypoxia-inducible factor 1ά (HIF1ά), was demonstrated by Takahashi and colleagues [174,175] in B16-BL6 melanoma cells. Twenty four hours after treatment with siRNA for β-catenin or HIF1ά mRNA levels were reduced to 25% and 35% of control values, respectively. After tumor cells were inoculated, intratumoral siRNA delivery and electroporation on days 7, 10 and 19 caused about an 80% decrease in tumor volume about 3 weeks after the initiation of treatment. Thus by suppressing β-catenin expression and thereby inhibiting Wnt signaling, which is important for expression of a number of genes important for angiogenesis, or by blocking expression of HIF1ά, which also directs the expression of genes involved in upregulation of angiogenic proteins, siRNA electrogene therapy can effectively inhibit tumor growth. However, it will be important to continue to investigate this method for cancer therapy by optimizing expression conditions, determining the most effective genes to silence for therapeutic benefit and to follow animals for longer periods of time to determine long term efficacy.

While therapies for melanoma have been designed to target specific molecules that are hallmarks of cancer and cytokine immunotherapy has been used to enhance immune responses, Nakai and co-workers [178] combined both approaches. Instead of targeting classic cancer hallmarks for their first target, they targeted microphthalmia-associated transcription factor (Mitf), which is involved in melanin synthesis as well as malignant transformation of melanocytes into melanoma. Thus, Mitf is a more specific cancer target for melanoma. Mitf is involved in a number of melanocyte and melanoma functions including not only pigmentation, but also survival, proliferation and melanoma progression. Mitf is a suspected melanoma oncogene that also induces HIF and VEGF. However, Mitf also has other reported functions that may be involved in cell cycle arrest, apoptosis and growth inhibition. Thus Mitf knockdown was shown to abate tumor growth. Previously, Nakai *et al*. [179] demonstrated that siRNA transfection of sequences corresponding to Mitf in mouse B16F10 melanoma tumors by both lipid-mediated and electroporation delivery downregulated Mitf and tyrosinase, which is involved in melanin synthesis, induced apoptosis and reduced tumor growth. By combining Mitf knockdown with IL-12 therapy, they targeted different anti-tumor mechanisms that could induce apoptosis as well as decrease angiogenesis, invasion and increase anti-tumor immunity.

### 4.5. Mechanisms for DNA Delivery to Cells and Tissues

Exactly how large molecules such as plasmid or naked DNA are transported across lipid bilayers, through the cellular milieu and across the nuclear membrane barrier to access the transcriptional machinery are still shrouded in some mysteries. Theoretical models predict that external electric fields increase the electrical conductivity of plasma membranes and when a critical threshold is reached (about 1 V) there is a transition from an insulating to a conductive state whereby lipid molecules are rearranged to form pores, or aqueous channels requiring a series of steps [87,180]. However, these pores have never been visualized and they cannot be as large as DNA plasmids, so DNA cannot pass through by simple diffusion [181]. Many studies of gene delivery define non-viral DNA vectors using DNA complexes with cationic liposomes, hydrophilic polymers or combinations of DNA with peptides or proteins [182,183,184]. Most of these non-viral DNA deliveries occur by endocytosis.

Several theories for electrogene delivery have been proposed and reviewed by Escoffre *et al*. [185]. Of greatest interest is increasing understanding of DNA-plasma membrane complex formation as an early and key step in electrogene transfer [186,187,188,189]. Data suggest that there are specific domains on plasma membranes that are competent to form stable DNA-lipid bilayer complexes in electroporated membranes [185,189]. Such complexes are similar in size to lipid rafts, which are cholesterol- and glycosphingolipid-enriched microdomain on the outer leaflet of plasma membranes that serve as platforms or recruitment centers involved in cell signaling and membrane trafficking [184,189]. Adenoviruses, adeno-associated viruses, retrovirus and liposomes have been shown to enter cells through clatherin-coated pits [184]. DNA-dendrimer complexes or dendriplexes appear to enter cells by mechanisms that require cholesterol and lipid raft integrity in endothelial cells [191]. This same group later found in HeLa and HepG2 hepatocellular carcinoma cells entry was more likely through dendrimer-DNA complexes with caveolin 1 [192]. Thus, mechanisms for delivery of these DNA complexes without electroporation were different in different cells. While a number of studies defining endocytosis pathways use general inhibitors, it will be important to investigate these mechanisms more specifically using specialized cell lines or knockdown strategies with dominant negative mutants, RNAi and/or knockout mice. Seternes and co workers [193] found that ^125^I-labeled plasmid DNA injected into the circulation of Atlantic cod was rapidly taken up by scavenger receptors in endocardial endothelial cells in the atrium and ventricles. This was confirmed in cultured atrial endothelial cells and in cultured cod head kidney leukocytes. This study also emphasizes another difficulty for therapeutic DNA expression since the plasmid DNA was degraded and not expressed in these cells. However, it might be expected that cells expressing and using scavenger receptors may be programmed for destruction of the cargo once internalized. 

Many of these results suggest that DNA delivery without electroporation may include endocytosis by one mechanism or another; the process does not seen to be random but likely at preferred sites or domains. However, the cell type and perhaps the nature of plasmids play roles in uptake mechanisms. For electrogene delivery of naked- and plasmid-DNA, it is important to ask if electric fields enhance inherent cellular mechanisms, induce unique membrane changes or some combination of the two. The concept of “pore” creation suggests unique features that may not be present, or present very infrequently, in non-permeabilized cells. On the other hand, DNA-membrane interactions in specific competent domains or well-defined permeabilized caps [189] suggest electric field interactions with inherent cellular mechanisms for electrogene delivery. Nevertheless, given that so many particulate macromolecules presented to cell membranes are subject to endocytosis, discussion of these mechanisms is not unreasonable. Cell entry pathways can be mediated by clatherin, non-clatherin, caveolae, non-caveolae endocytosis pathways as well as phagocytosis and macro-pinocytosis [184]. Recruitment of any of these pathways is likely to depend upon the cell type and nature (size, sequence, others) of DNA constructs. Because the mechanism of intracellular transport begins with DNA binding to cell membranes, the cell entry pathway may determine the pathways for cytoplasmic trafficking, endosmolysis, and nuclear entry mechanisms. Thus, the cell entry mechanisms could be fundamental to understanding subsequent mechanisms for electrogene delivery and therapeutic expression. It is clear that the plasma membrane is only the first of several barriers for DNA to reach the transcription machinery. DNA must traverse the landscape of the cytosol, which is not a sea of physiological salt, but a crowded topography of cytoskeletal structures presenting navigation barriers. It has been hypothesized that microtubular structures are altered by the electric fields, especially those near the plasma membrane [185,194]. It is also known that the cytoskeleton provides a highway for vesicular transport mediated by molecular motors as exemplified by dynein motors transporting vesicles budding from clatherin-mediated pathways along microtubules. 

In cases of DNA containing vesicles, the question arises as to how DNA escapes endocytotic vesicles, a major barrier to efficient gene transfer. These considerations may be excluded for instance when DNA is transported across the plasma membrane without vesicles as it is in planar lipid bilayers [195]. These data indicate that DNA is electrophoretically pulled through the porous zones in the planar membrane. The cargo in transported vesicles are often transported to lysosomes and degraded by proteases and several mechanisms for endosomolysis have been considered [182]. DNA is designed to withstand all measures of degradation, but DNAses are its major nemesis. 

Another barrier to expression is the nuclear membrane. It is known that gene expression is greatest for cells that are dividing when the nuclear membrane is degraded, so access to transcription machinery is limited. As indicated earlier, delivering IL-12 and IL-18 using an Epstein-Barr-based plasmid included features that enhance expression potential [154]. As electrogene delivery mechanisms are better understood, it may be possible to engineer specialized sequences to enhance therapeutic outcome. However, as DNA plasmid constructs become larger, their delivery and expression becomes encumbered by electroporation.

## 5. Conclusions

Bioelectrics is a new field that provides novel strategies for treating melanoma and other cancers. Using nanosecond pulsed electric fields provides a means to eliminate melanoma tumors in mice [30,55,59] by inducing characteristics typical of apoptosis in B16F10 melanoma cells [54] and tumors in mice [55]. It directly targets two cancer hallmarks, apoptosis evasion and sustained angiogenesis, and through the latter one suggests a third hallmark, invasion and metastasis. These are well-defined therapeutic objective for treating melanoma and other cancers [54]. In another Bioelectrics domain, applications of micro- and milli-second pulses have proven effective for eliminating melanoma by a number of mechanisms revolving around electrogene transfer. Electrogene therapy is a simple, safe and effective method to deliver genes to tumor cells and tissues. It has proven effective for cancer hallmarks including evasion of apoptosis, sustained angiogenesis (invasion and metastasis) and evasion of immune surveillance. Its effectiveness in clinical trials [159] establishes it as a major method for gene therapy. As perhaps the most effective means of non-viral gene delivery to date, it is anticipated that it will find continued therapeutic successes with delivery of other genes and/or RNAi that will interfere with tumorigenesis, making a greater impact on cancer therapies than previous modalities.
